# Duodenal Amyloidosis Inducing Malabsorption in Hepatitis B-Related Liver Cirrhosis: A Rare Case Presentation

**DOI:** 10.7759/cureus.80147

**Published:** 2025-03-06

**Authors:** Chanchal Kumar Ghosh, Aditi Sarker, Sumona Islam, Nafizul Islam, Prodipta Chowdhury, Mahjabin Islam, Amit Bari

**Affiliations:** 1 Department of Gastroenterology, Bangabandhu Sheikh Mujib Medical University (BSMMU), Dhaka, BGD; 2 Department of Gastroenterology, Bangladesh Medical College Hospital, Dhaka, BGD; 3 Department of Gastroenterology, Keshabpur Upazila Health Complex, Keshabpur, BGD; 4 Department of Endocrinology, Bangabandhu Sheikh Mujib Medical University (BSMMU), Dhaka, BGD; 5 Department of Nephrology, Kidney Foundation Hospital and Research Institute, Dhaka, BGD

**Keywords:** chronic hepatitis b, gastrointestinal amyloidosis, liver cirrhosis, malabsorption syndrome, secondary amyloidosis

## Abstract

Chronic hepatitis B virus (HBV) infection is a major cause of liver cirrhosis worldwide. While hepatic complications are well-documented, extra-hepatic manifestations such as secondary amyloidosis are less common and can lead to diverse and complex clinical outcomes. Here, we present the case of a 60-year-old male with a history of chronic hepatitis B who progressed to liver cirrhosis and presented with chronic, unexplained, intractable diarrhea unresponsive to standard treatments. He also experienced significant unintentional weight loss and generalized weakness. Further investigations revealed amyloid deposition in the duodenum, confirming secondary amyloidosis affecting the gastrointestinal tract as the cause of malabsorption. Persistent diarrhea and malabsorption necessitated nutritional support and symptomatic management. This case highlights the rare association between chronic liver disease (CLD), secondary amyloidosis, and malabsorption syndrome, emphasizing the importance of clinical suspicion for timely diagnosis. Early recognition and prompt intervention are crucial to managing these complex cases, improving outcomes, and preventing further complications.

## Introduction

Hepatitis B virus (HBV) infection is a significant global health concern, affecting millions worldwide. Chronic hepatitis B infection is associated with numerous complications, with liver cirrhosis being one of the most common and severe outcomes [1]. While cirrhosis itself is a major cause of morbidity and mortality, it can also predispose patients to the development of secondary conditions, some of which are rare and poorly understood.

Amyloidosis, characterized by the abnormal extracellular deposition of protein aggregates in various organs, is a rare but potentially debilitating complication of chronic liver disease (CLD) [2]. Hepatitis B-related cirrhosis leading to secondary amyloidosis and subsequent malabsorption syndrome is an uncommon and underreported manifestation [3].

In this case report, we present a rare and unique instance of longstanding chronic hepatitis B-related cirrhosis complicated by secondary amyloidosis, resulting in malabsorption syndrome. Recognizing the association between chronic HBV infection, cirrhosis, amyloidosis, and malabsorption syndrome is crucial for clinicians to effectively identify and manage these complex presentations.

## Case presentation

A 60-year-old male with a history of chronic hepatitis B infection presented to the gastroenterology department with chronic diarrhea, unexplained weight loss of 15 kg over the past 6 months, and generalized weakness. The watery diarrhea occurred 6-7 times a day, sometimes with mucus, was associated with abdominal cramping, and worsened after meals. Despite maintaining his usual dietary intake, the patient reported a significant decrease in appetite and energy levels.

The patient had been diagnosed with chronic hepatitis B infection, which had progressed to cirrhosis of the liver. On examination, he appeared pale and emaciated, with glossitis, angular stomatitis, bipedal edema, dry ichthyotic skin, and lusterless hair. Abdominal examination revealed splenomegaly with ascites.

Given his clinical presentation, a thorough diagnostic workup was performed.

The laboratory investigations (Table 1) revealed a low hemoglobin level of 8.2 g/dL, elevated erythrocyte sedimentation rate (ESR), and thrombocytopenia. Liver function tests showed hypoalbuminemia (serum albumin 15 g/L), with prolonged prothrombin time (PT) and an International Normalized Ratio (INR) of 1.25. Notably, the patient's serum ferritin and ceruloplasmin levels were elevated, while serum calcium was low. Nutritional deficiencies were evident, with low levels of vitamin D and folate. The patient's stool tests and anti-TTG antibody were negative for celiac disease.

**Table 1:** Laboratory investigations. ESR: Erythrocyte Sedimentation Rate; S: Serum; PT: Prothrombin Time; INR: International Normalized Ratio; ALT: Alanine Aminotransferase; AST: Aspartate Aminotransferase; HBc: Hepatitis B Core Antibody; FBS: Fasting Blood Sugar; ABF: After Breakfast Fasting; SAAG: Serum-Ascites Albumin Gradient; ADA: Adenosine Deaminase; AFB: Acid-Fast Bacilli; MT: Mantoux Test (tuberculin skin test); TSH: Thyroid-Stimulating Hormone; HIV: Human Immunodeficiency Virus; RA: Rheumatoid Arthritis; Anti-CCP: Anti-Cyclic Citrullinated Peptide; ANA: Antinuclear Antibody; Anti-TTG: Anti-Tissue Transglutaminase; OBT: Occult Blood Test; R/M/E: Routine, Microscopy, Examination; BUN: Blood Urea Nitrogen; LDH: Lactate Dehydrogenase; CRP: C-reactive Protein; HBV: Hepatitis B Virus.

Investigations	Patient	Reference Value
Hemoglobin (g/dL)	8.2	13-15
ESR (mm in 1st hour)	25	0-20
WBC (/cumm)	3,500	4,000-11,000
Platelet (/cumm)	120,000	150,000-450,000
S. Albumin (g/dL)	15	35-50
PT (seconds)	14.9	12-14
ALT (U/L)	22	8-56
AST (U/L)	26	5-40
Anti HBc (Total)	Positive	-
S. Ferritin (ng/mL)	214	50-450
S. Ceruloplasmin (mg/L)	20	20-40
S. Folate (ng/mL)	4.6	2.7-17
Vit. B12 (pg/mL)	459	200-900
S. Calcium (mg/dL)	8.2	8.5-10.5
S. Magnesium (mg/dL)	1.5	1.7-2.2
Vit-D (ng/mL)	14.2	20-50
Zinc (µg/dL)	45.6	70-120
S. Creatinine (mg/dL)	1.09	0.6-1.2
S. Electrolytes (mmol/L)	Na 135, K 4.6	Na 135-145, K 3.5-5
Blood Sugar (mmol/L)	FBS: 3.5, 2 hrs ABF: 5.6	FBS: 3.9-5.5, 2 hrs ABF: 7.8-11
Ascitic Fluid Study	SAAG: 15.2 g/L, ADA: 10.2 U/L, Protein 10.1 g/L, AFB and GeneXpert negative	-
MT	Negative	-
TSH (uIU/mL)	2.89	0.5-5
HIV-1 & 2	Negative	-
RA & Anti-CCP	Negative	-
ANA	Negative	-
Anti-TTG antibody	Negative	-
Stool OBT	Negative	-
Stool R/M/E	Normal	-
S. Protein Electrophoresis	Polyclonal Gammopathy	-

**Figure 1:**
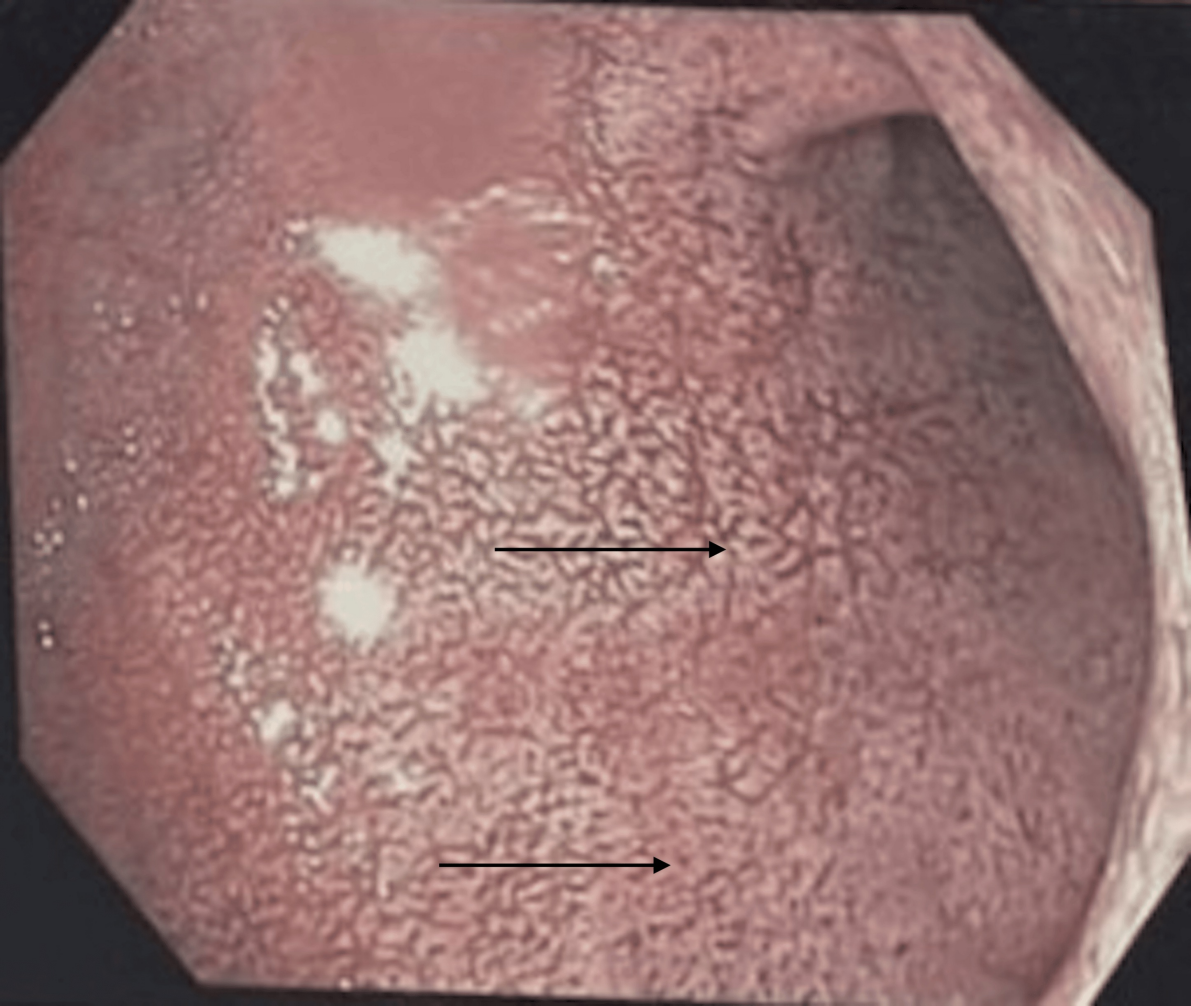
Upper GI endoscopy showing patchy white plaques in the first part of the duodenum. GI: Gastrointestinal.

Ultrasound of the abdomen revealed hepatosplenomegaly with coarse hepatic parenchyma and significant ascites, suggestive of CLD. Upper GI endoscopy showed patchy white plaques and prominent villi throughout the 1st and 2nd parts of the duodenum (Figure 1).

**Figure 2:**
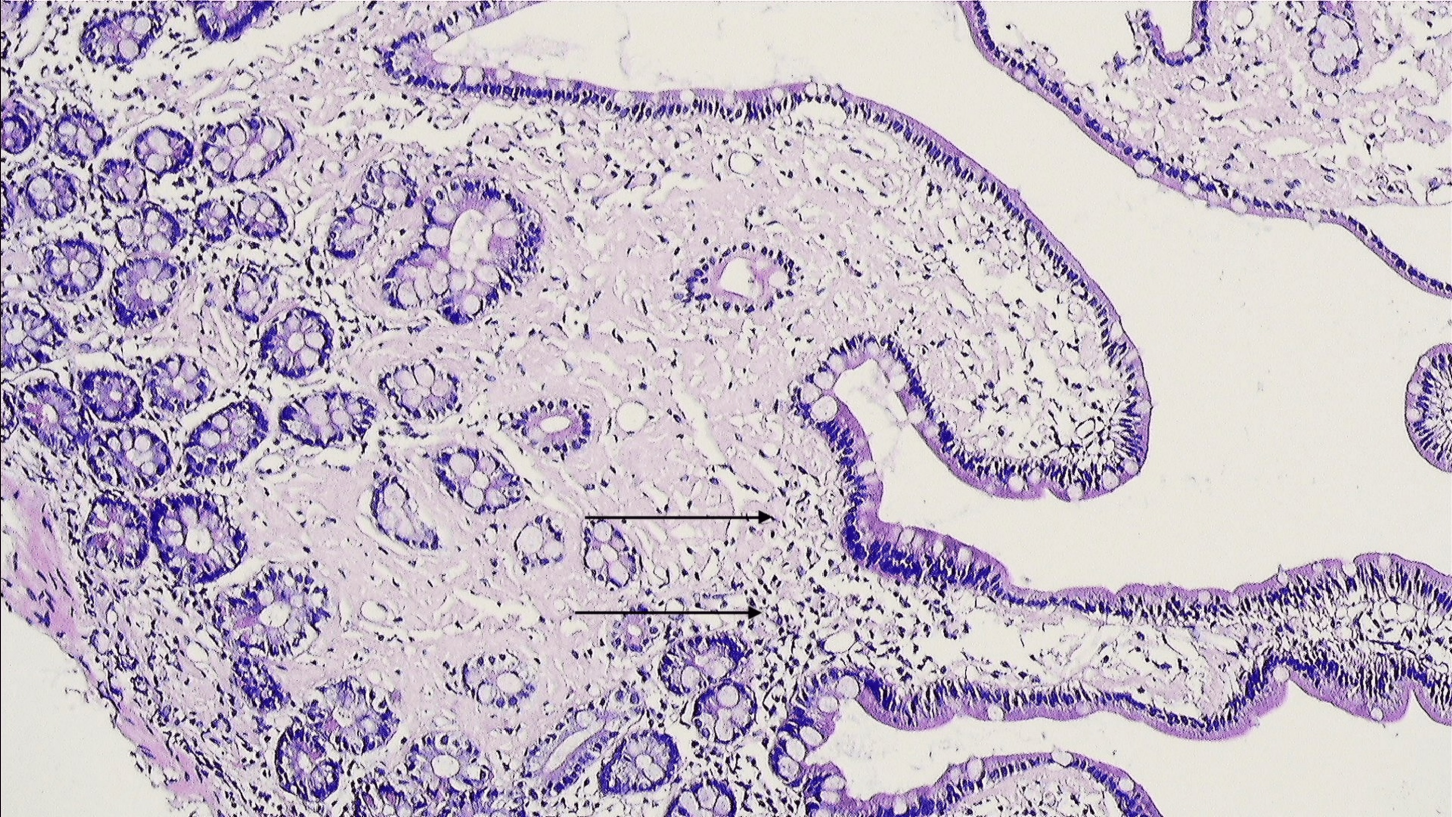
Histopathology showing intraepithelial lymphocytes without crypt hyperplasia.

Microscopic examination of duodenal tissue demonstrated short and broad villi, with no crypt hyperplasia or increased intraepithelial lymphocytes (Figure 2).

**Figure 3:**
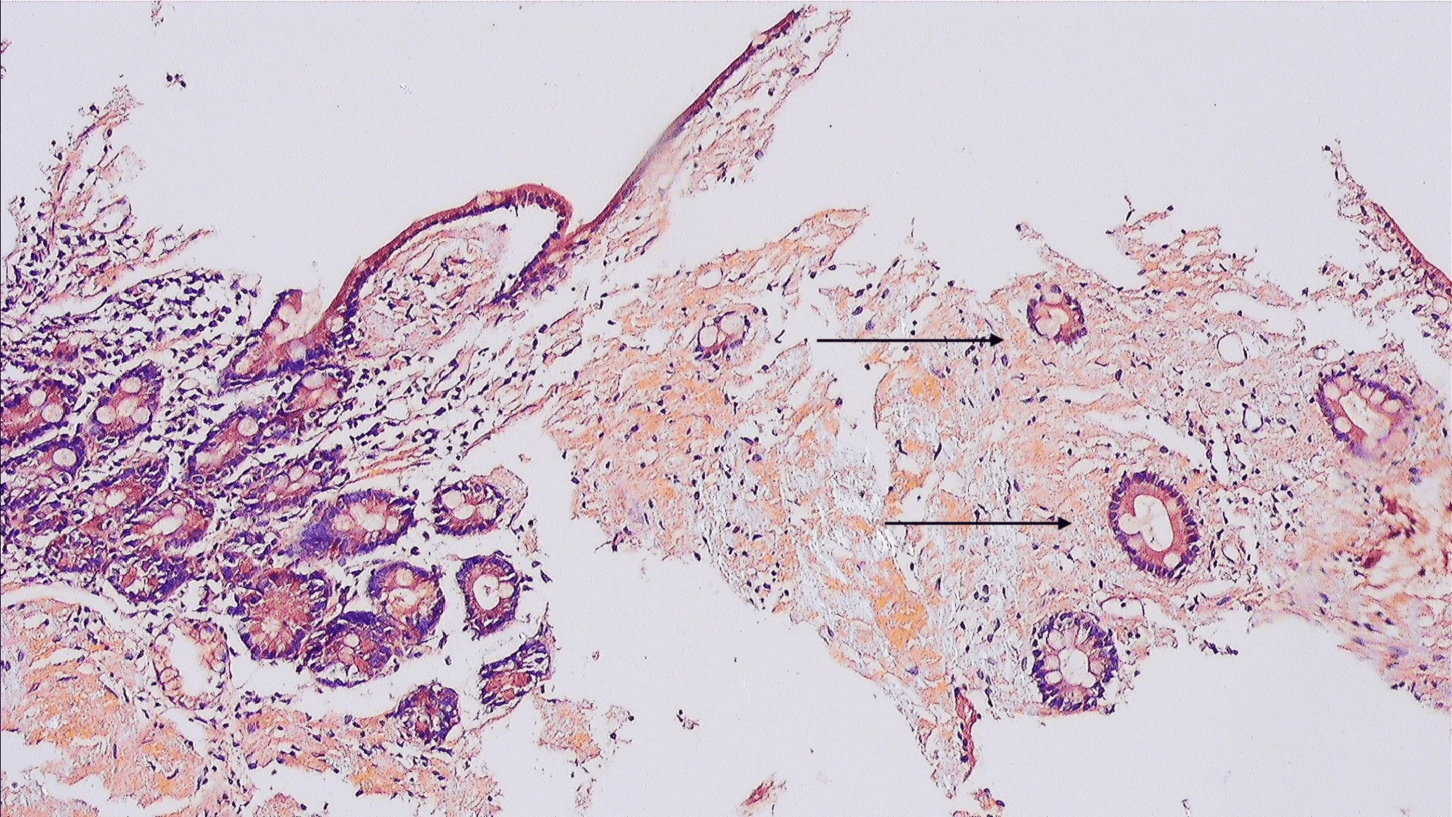
Congo red stain showing eosinophilic positive material.

However, the villi revealed eosinophilic positive material in the core, which was Congo red positive (Figure 3).

Under polarizing microscopy, apple-green birefringence was observed, confirming the presence of amyloid deposits.

The patient was diagnosed with hepatitis B-related cirrhosis complicated by secondary amyloidosis, which led to malabsorption syndrome. He was started on supportive therapy for liver disease, including lactulose and diuretics for ascites and hepatic encephalopathy. Additionally, management for malabsorption syndrome was initiated, which included dietary modifications and nutritional supplementation.

The patient continues to be monitored closely for any complications related to his liver disease and amyloidosis, with ongoing adjustments to his management plan based on his clinical progress.

## Discussion

Systemic amyloidosis is a group of disorders characterized by the deposition of insoluble proteins, known as amyloid, in tissues and organs. The most common form is light chain amyloidosis (AL), caused by the accumulation of misfolded immunoglobulins. Another form, AA amyloidosis, results from the deposition of amyloid A protein, which is generated from serum amyloid A, an acute-phase reactant. While amyloidosis commonly affects organs like the kidneys, heart, and nervous system, its involvement in the GI tract remains relatively rare [4].

GI amyloidosis, which refers to amyloid deposition in the GI tract, is characterized by a variety of symptoms including diarrhea, malabsorption, weight loss, and steatorrhea. Histopathological confirmation through biopsy is essential for diagnosis, yet it is an uncommon finding. GI amyloidosis has been reported in approximately 60% of patients with AA amyloidosis, though only in 8% and 1% of patients with AL amyloidosis, based on biopsy and clinical diagnoses, respectively [5]. The involvement of the GI tract in systemic amyloidosis can be seen through various endoscopic findings, including a fine granular appearance or polypoid protrusions, particularly in the duodenum. Different types of amyloid deposits (AL, β2M, and ATTR) tend to deposit submucosally, whereas AA amyloid is more commonly found in the superficial mucosal layer [6,7].

The small intestine is the most common site of amyloid deposition, affecting approximately 30% of amyloidosis patients. Among these, malabsorption is observed in 8.5% of those with AL amyloidosis and 2.3% of those with AA amyloidosis [8]. AA amyloid deposition in the mucosa can cause friability, erosions, and the clinical presentation of diarrhea and malabsorption. In patients with amyloidosis, malabsorption can result from mucosal infiltration, pancreatic insufficiency, or bacterial overgrowth. This can lead to weight loss, which has been reported to be as high as 13.6 kg in some cases [8,9].

Secondary amyloidosis, associated with chronic inflammatory disorders and microbial infections, is primarily caused by the deposition of AA amyloid. Chronic hepatitis B infection is an uncommon cause of systemic AA amyloidosis, though viral infections, including hepatitis B and C, have been reported in a few cases. Hepatitis B infection has been linked to nephrotic syndrome due to renal AA amyloidosis, as observed in a 13-year-old patient [10]. Additionally, a systematic review has shown strong disease associations between systemic AA amyloidosis and chronic viral infections, including hepatitis B and C [11]. However, the co-occurrence of hepatitis B-related cirrhosis and GI amyloidosis has not been widely reported.

While amyloidosis rarely presents with hepatic failure, it should be considered in patients with signs of liver decompensation, particularly in the context of chronic infections such as hepatitis B. Early identification of amyloidosis in such patients is crucial, as it can have serious consequences if left unrecognized [11].

## Conclusions

This case highlights the complex interplay between chronic hepatitis B infection, cirrhosis of the liver, amyloidosis, and malabsorption syndrome. The presence of amyloidosis in the setting of liver cirrhosis presents a diagnostic challenge and emphasizes the need for comprehensive investigations in patients with unexplained gastrointestinal symptoms. Given the rarity of this association, clinicians must maintain a high degree of suspicion and employ appropriate diagnostic tools to confirm the presence of amyloidosis. Early recognition and intervention are crucial to improving patient outcomes, enhancing their quality of life, and preventing further complications. As understanding of the pathophysiology and clinical manifestations of these overlapping conditions evolves, further research is needed to establish optimal management strategies and therapeutic approaches.
